# Responsibility Voids and Cooperation

**DOI:** 10.1177/0048393118767084

**Published:** 2018-03-29

**Authors:** Hein Duijf

**Affiliations:** 1 Utrecht University, Utrecht, The Netherlands

**Keywords:** responsibility voids, cooperation, practical reasoning, team reasoning, uncertainty

## Abstract

Do responsibility voids exist? That is, are there situations in which the group is collectively morally responsible for some outcome although no member can be held individually morally responsible for it? To answer these questions, I draw a distinction between competitive and cooperative decision contexts based on the team-reasoning account of cooperation. Accordingly, I provide a reasoning-based analysis of cooperation, competition, moral responsibility, and, last, potential responsibility voids. I then argue that competitive decision contexts are free of responsibility voids. The conditions for the existence of responsibility voids in cooperative decision contexts depend on the type of uncertainty the group faces, either external or coordination uncertainty.

## 1. Introduction

Do responsibility voids exist? That is, are there situations in which the group is collectively morally responsible for some outcome although no member can be held individually morally responsible for it? In committee decision making, this yields the question of whether there is a voting procedure and a combination of votes such that none of the members is an appropriate target of moral criticism, regardless of their involvement in bringing about a certain state of affairs. This issue is important for at least two reasons. First, committee decision making is one of the pillars of democratic institutions, and responsibility voids may highlight a flaw in democratic regulations. Second, responsibility voids have recently been used to justify the need for corporate responsibility, as opposed to individual responsibility, to dissolve the deficit in the moral accounting books (Copp 2006; Pettit 2007). Philip Pettit (2007, 196-97) writes,

Let group agents be freed from the burden of being held responsible, and the door will open to abuses: there will be cases where no one is held responsible for actions that are manifestly matters of agential responsibility.

The philosophical literature on collective moral responsibility concerns three interrelated questions: (a) Can we make sense of *collective* moral responsibility? (b) How does collective moral responsibility *distribute* over its members? (c) What is the *value* of collective moral responsibility? In this paper, I largely focus on the second question and enquire whether cases exist in which a group is collectively morally responsible for some outcome although no member can be held morally responsible for it.^1^ The question of the members’ individual moral responsibility will depend on the facts of the particular case. By providing conditions that must be met for responsibility voids to exist, I show *which* facts matter.

Whether collective moral responsibility distributes to its members is a matter of much debate. For example, collective action problems have been examined before in terms of voting paradoxes (Pettit 2007), causal contributions, and information states of the involved individuals (Braham and van Hees 2012), and the nature of the intentions of the participants (Kutz 2000). Inspired by the team-reasoning account of cooperation (Bacharach 2006), I adopt a new take on the problem and propose to use the distinction between cooperation and competition to assess the moral responsibility of individuals in collective action contexts.

Team-reasoning theorists rely on different modes of *reasoning* to contrast cooperation and competition. Simply stated, an individual agent faces a competitive decision problem if and only if she reasons individualistically; she faces a cooperative decision problem if and only if she team reasons. To address typical collective action problems, the team-reasoning paradigm needs to be refined to what I call *participatory reasoning*. The details of these reasoning methods need not worry us at present as they will be explicated in the following sections.

In decision theory, it is common to distinguish between making decisions under certainty, risk, and uncertainty.^2^ In line with this distinction, I will show that the outcome of participatory reasoning depends on two types of uncertainty. *External uncertainty*: this is where the collective outcome is influenced by expectations regarding external factors. For example, these external factors may include what your opponents are doing, or they may concern physical properties of the world, such as whether it is currently raining. *Coordination uncertainty*: this is where the collective outcome is influenced by expectations regarding in-group coordination. For instance, it may be that there is no correlation device available to successfully coordinate the members’ actions. This distinction will be relevant for assessing the conditions for the existence of responsibility voids in cooperative decision contexts.

To apply the reasoning-based distinction between cooperation and competition, offered by the team-reasoning literature, to collective action problems, I need to provide a *reasoning-based* framework of moral responsibility, or at least a sketch thereof. Roughly stated, to assess whether an individual is responsible for a certain outcome, I study the practical reasoning that has led to her decision.

Based on my reasoning-based framework of moral responsibility for outcomes, I can assess the possibility of responsibility voids. Given the distinction between competitive and cooperative decision contexts, my arguments are twofold. First, I argue that competitive decision contexts never yield responsibility voids. In such contexts, there cannot be collective moral responsibility for a certain outcome without there being a morally responsible member. Second, I argue that cooperative decision contexts potentially host responsibility voids. To clarify this point, I illustrate these responsibility voids for both types of uncertainty: external uncertainty and coordination uncertainty. I argue that both types of uncertainty host potential responsibility voids, although the conditions for the existence of such voids differ.

The paper is set out as follows. After some initial ground-clearing with regard to the literature on voting paradoxes in section 2, I provide a sketch of a reasoning-based framework of moral responsibility using practical-reasoning schemas in section 3. In section 4, I discuss the team-reasoning account of cooperation using practical-reasoning schemas, and argue that team reasoning needs to be refined to participatory reasoning to address collective action problems. In section 5, I reconsider the existence of responsibility voids and argue for my two main claims: competitive decision problems are free from responsibility voids; cooperative decision problems potentially host responsibility voids, but the conditions for the existence of such voids differ depending on the type of uncertainty. I conclude with a brief discussion of the implications for corporate responsibility and democratic decision making.

## 2. Some Ground-Clearing

**Figure 1.**
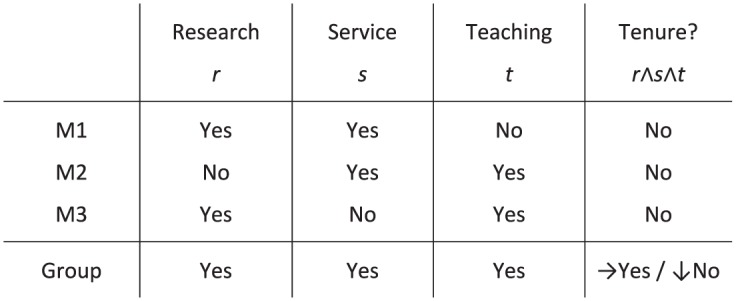
The discursive dilemma.

A prominent problem in the philosophical literature on collective decision making is the *discursive dilemma*, which is also known as the doctrinal paradox in legal theory (Chapman 1998; Kornhauser 1992; the former introduced the term ‘*doctrinal paradox*’). To illustrate the dilemma, suppose a committee of academics, consisting of M1, M2, and M3, is deciding on whether to award tenure to Mr. Borderline. Imagine the university’s tenure policy requires excellence in research, service, and teaching. Suppose the committee members are to decide on the tenure by first voting on each of these fields of competence, then aggregating their votes on each of these fields by majority, and finally deriving the collective decision on the tenure in line with the university’s rules. If the members vote in accordance with Figure 1, then it turns out that they collectively decide to award tenure even though they are unanimously opposed.

According to Philip Pettit, this scenario illustrates that there is a dilemma involving which collective decision procedure to endorse. First, the committee members could adopt the procedure stated in the example, which yields awarding tenure. This is commonly referred to as the premise-driven collective decision procedure. Second, the committee members could aggregate their judgments on the conclusion, which would yield refusing tenure. This is commonly referred to as the conclusion-based collective decision procedure. Pettit (2001) connects this dilemma to the literature on deliberative democracy and then presents arguments from republican theory that suggest that a premise-based collective decision-making procedure should be adopted to account for the reasons-responsiveness of the collective. Pettit’s perspective of the dilemma is different from the employees’ personal purview and is best called an *external perspective*. As designers, we may ask what would be a sensible way of aggregating the individuals’ judgments into a collective judgment.^3^ However, it is important to note that similar scenarios can be given for *any* collective decision procedure that satisfies some intuitive constraints (List and Pettit 2002, Theorem 1).^4^

The perspective I adopt in this paper is therefore different. Assume that Mr. Borderline actually does not satisfy the conditions for tenure. To study potential responsibility voids, it is vital to ask whether any of the members is an appropriate target of moral criticism for the incorrectly awarded tenure. Instead of asking how we may sensibly aggregate individual judgments, I therefore ask whether, under the given circumstances, any of the committee members can be held responsible for awarding tenure. My perspective may be called an *internal* decision-theoretical perspective. It includes the study of how an individual should take a personal decision *given a particular collective decision procedure*. Game theory offers a useful framework for studying individual decision making in such interdependent decision problems, that is, scenarios in which the collective outcome depends on the interaction of several individuals.^5^

## 3. Reasoning-Based Moral Responsibility

To apply the reasoning-based distinction between cooperation and competition (section 4) to collective action problems, it is instructive to sketch an outline of a reasoning-based framework for moral responsibility. For simplicity’s sake, I focus on blameworthiness for outcomes rather than praiseworthiness. Although I think it is useful and important to further develop this reasoning-based framework, for my present purposes, this sketch will suffice to highlight its main features and, roughly, its application.

To make a start with constructing an account of moral responsibility that relies on practical reasoning, I aim to use practical-reasoning schemas. Following Gold and Sugden (2007), various forms of practical reasoning can be characterized by *schemas* of practical reasoning.^6^ Such schemas illustrate how premises describing the decision context and describing what the agent seeks to achieve should be used to decide which action should be taken. In logic, a valid rule of inference illustrates how premises should be used to derive conclusions. Analogously, the fundamental idea is that a practical-reasoning schema infers conclusions about what the agent ought to do from premises that include propositions about what the agent is seeking to achieve. For instance, when reasoning individualistically, the agent first considers the individual actions available to her, assesses these individual actions in terms of their consequences, and then finds the individual action that best furthers her interests.

**Figure 2.**
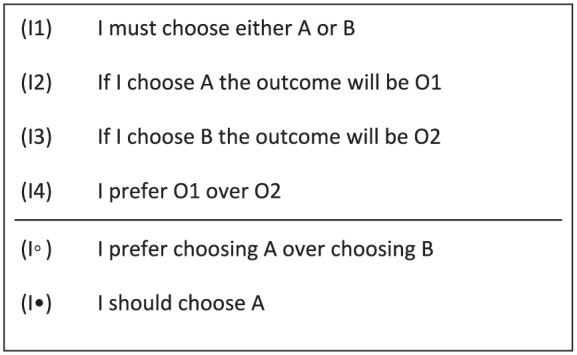
Individual-reasoning schema: (I•) states the conclusion; (I◦) states an intermediate conclusion; and (I1)–(I4) state the premises.

Figure 2 depicts a reasoning schema that characterizes individually instrumental reasoning.^7^ The first premise (I1) expresses the available or eligible individual actions. The two premises (I2) and (I3) state that the available individual actions are evaluated on the basis of their possible consequences. Premise (I4) describes what the agent seeks to achieve in terms of a preference over outcomes. The individual-reasoning schema emphasizes that the personal preferences over outcomes (I4) are lifted to preferences over the available individual actions (I◦): since I prefer O1 over O2, I prefer choosing A over choosing B. Finally, this lifted preference delivers the conclusion of practical reasoning: because I must choose one of them, this entails that I should choose A (I•).

It may be helpful to note that this reasoning schema can be straightforwardly adopted in decision contexts that involve risk, that is, in cases where “each action leads to one of a set of possible outcomes, each outcome occurring with a known probability” (Luce and Raiffa 1957, 13). To see this, imagine an individual agent playing a game of chess against a sophisticated computer. For simplicity’s sake, let us imagine that she seeks to win the game. To decide on her next move, the individual agent has to consider the individual actions available to her. Plausibly, only individual actions that abide by the rules of chess are admissible for consideration. She then needs to assess the expected consequences of her eligible individual actions. Say she expects that choosing 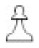
**e4** has a 75% chance of leading to victory, whereas choosing 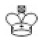
**d6** only has a 30% chance of doing so. Consequently, assuming she endorses something like expected utility maximization, she prefers choosing 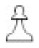
**e4** over choosing 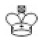
**d6** because it has the best chance of leading to victory. Finally, this will lead her to conclude that she should choose 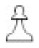
**e4**.

We can see that the individual-reasoning schema can certainly fail to deliver a determinate solution.^8^ That is, if individual reasoning delivers *Z* as the set of recommended individual actions, then, on the one hand, one may think that each individual action *A* ∈ *Z* is permitted, or admitted, by individual reasoning. On the other hand, one may think that the individual agent is required to choose one of the individual actions in *Z*.^9^

Since individual instrumental reasoning seems to naturally cohere with consequentialist moral reasoning, one may ask whether it can be generalized to incorporate deontological moral reasoning. This issue cannot be settled here, but I wish to add two comments. First, Portmore (2007, 39) gives a recipe for consequentializing nonconsequentialist theories: “Take whatever considerations that the nonconsequentialist theory holds to be relevant to determining the deontic status of an action and insist that those considerations are relevant to determining the proper ranking of outcomes.” For example, if a deontological theory holds that it is wrong for an agent to violate someone’s rights even for the sake of minimizing rights violations, overall, then the corresponding consequentialist theory needs to hold that any outcome where the agent violates someone’s rights ranks lower than any outcome where the agent does not do so, even if the agent’s violation would prevent others from committing more rights violations. This suggests that if deontological theories are consequentializable, then deontological moral reasoning can be incorporated by amending the preferences accordingly. Second, Dietrich and List (2017, 456) provide a reason-based decision-theoretic framework that can represent virtually any moral theory by way of a so-called “reasons structure,” “which encodes the theory’s answer to the two-part question of which properties matter and how they do so.” A moral theory’s associated reasons structure can readily be plugged into the individual-reasoning schema.^10^ Instead of assessing the available options in terms of their consequences (as in (I2) and (I3)), they would be assessed in terms of their properties. These associated sets of properties are then compared using the reason structure’s weighing relation, or defeat relation, rather than using a preference relation (as in (I4)). In this way, the amended individual-reasoning schema seems to be able to accommodate virtually any moral theory.

Reasoning schemas highlight that there may be different reasons for holding an individual agent responsible for a certain outcome. As mentioned before, I will focus on moral blameworthiness for outcomes. The intuitive idea is that moral blameworthiness requires or relies on bad intentions or culpable ignorance, or at least on moral faultiness.

To illustrate how the individual-reasoning schema may help develop a reasoning-based framework of responsibility for outcomes, let us consider a case of committee decision making. A hiring committee consists of three members, Marie, Malcolm, and Myra, and it has to make a choice about which of three candidates, Alice, Bill, and Charlie, is to join the company board. Committee decisions are made by majority, and with Marie, the chairperson, breaking ties. The committee members vote for Alice, Bill, and Charlie, respectively. The result is that Alice wins. Let us suppose that Alice turns out to be a bad candidate for the company board.

Is Malcolm blameworthy for the outcome that Alice wins? I provide three complementing analyses of this scenario; each emphasizes a particular type of concern, and each is associated with some premises of the individual-reasoning schema.^11^ First, Malcolm’s preferences may have been such that it was irrelevant for him whether Alice wins. It may, for instance, have been the case that he randomly decided on his vote. In this case, Alice’s winning played no role in his practical reasoning. It seems implausible to say that Malcolm is morally blameworthy for the result that Alice wins if it played no role in his practical reasoning. To assess Malcolm’s responsibility in more detail, we need to ask whether it was reasonable for him to be indifferent about Alice’s winning. He would be morally blameworthy for Alice’s winning only if it was unreasonable for him to be so indifferent.^12^ If it was unreasonable, then this would illustrate a moral failure in premise (I4) of the individual-reasoning schema, which describes what Malcolm seeks to achieve.

Second, Malcolm’s preferences may have been such that he sought to let Alice lose. In such a case, Alice’s losing played a vital role in his practical reasoning. How could voting for Bill have been permitted by individual reasoning? For example, he could have voted as he did if he expected that Myra would also vote for Bill. In that case, he would expect that his vote would yield Bill’s winning. However, his expectation regarding Myra’s vote was wrong, leading to Alice’s winning. This illustrates a moral failure in premise (I2) or (I3) of the individual-reasoning schema, which describes Malcolm’s expectation regarding the consequences of voting for Bill. So we may say that Malcolm is morally blameworthy for Alice’s winning: his false expectation led to Alice’s winning. To assess Malcolm’s responsibility in more detail, we need to ask whether it was reasonable for him to expect that Myra would vote for Bill. Simply stated, his moral responsibility for Alice’s winning could be undermined if his expectations were reasonable.

Third, Malcolm may have considered only a subset of the viable options. For instance, he may have thought that casting a vote for Charlie was not an eligible option for him. In this case, Malcolm basically only considered voting for Bill or Alice, then reasoned so as to minimize the expectation that Alice wins, and finally decided to vote for Bill. It then seems plausible to say that Malcolm is not morally blameworthy for Alice’s winning. To refine the assessment of moral responsibility, we may ask whether it was reasonable for him to think that voting for Charlie was not an eligible option. Simply stated, he would be morally blameworthy for Alice’s winning if he should have known that voting for Charlie was, in fact, an eligible option. The blameworthiness would correspond to a moral failure in premise (I1) of the individual-reasoning schema, which states the eligible individual actions.^13^

## 4. Cooperation

To characterize competition and cooperation, I rely on the team-reasoning account of cooperation (Bacharach 2006; Gold and Sugden 2007; Sugden 2000). The team-reasoning account of cooperation appeals to the *reasoning method* by which an individual agent reasons about what to do.^14^ An individual agent engaged in team reasoning “works out the best feasible combination of actions for all the members of her team, then does her part in it” (Bacharach 2006, 121). In this way, team reasoning offers an alternative to standard individualistic reasoning in collective action problems. The core idea of team reasoning is that an individual asks herself “What should *we* do?” rather than “What should *I* do?” Team reasoning hence relies on a we-perspective.^15^

**Figure 3.**
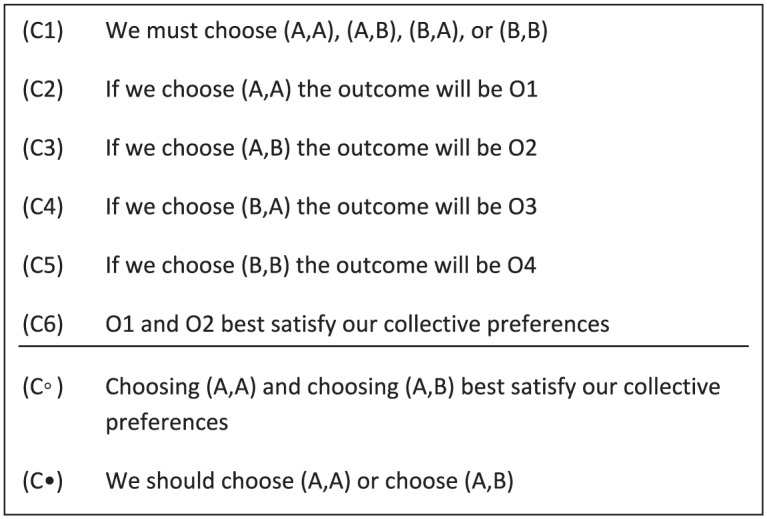
Collective-reasoning schema: (C•) states the conclusion; (C◦) states an intermediate conclusion; and (C1)–(C6) denote the premises.

Before we discuss team reasoning, note that the individual-reasoning schema can be straightforwardly amended for groups: the collective-reasoning schema in Figure 3 illustrates that the preferences over outcomes are lifted to the available group actions, and the schema highlights that it may fail to deliver a determinate solution.^16^ The only difference with the individual-reasoning schema is the level of agency: in the individual-reasoning schema, individual preferences over outcomes are lifted to preferences over individual actions; in the collective-reasoning schema collective preferences over outcomes (C6) are lifted to preferences over group actions (C◦). Given the symmetry, the collective-reasoning schema is valid if and only if the individual-reasoning schema is.^17^

It is helpful to emphasize that collective reasoning relies on group preferences. In line with the team-reasoning literature (see Bacharach 2006; Sugden 2000), I assume that group preferences are given.^18^ As such, my study is independent of any specific account of group preferences.

**Figure 4.**
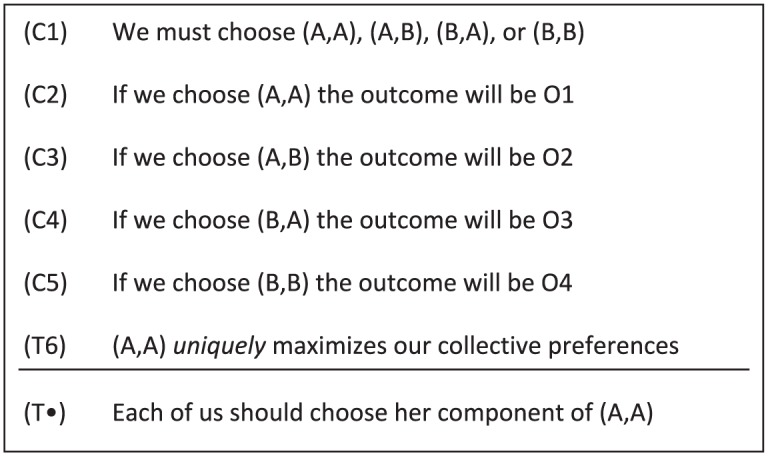
Team-reasoning schema: (C1)–(C5) and (T6) state the premises, and (T•) states the conclusion.

Proponents of team reasoning have adopted the reasoning schema depicted in Figure 4 for group members adopting a we-perspective. It may be helpful to note that there is a subtle difference between team reasoning and team-directed reasoning. For a given group *G*, Sugden (2000, 195; emphasis added) writes, “Suppose the following two conditions are satisfied. First, each individual *i* ∈ *G* engages in *team-directed reasoning* with respect to *G* and [the group’s preferences]. Second, each individual *i* ∈ *G* has full team confidence with respect to *G* and [the group’s preferences]. Then . . . the team engages in *team reasoning*.”^19^ Because I will not study the network of common beliefs required for full team confidence, my study can be viewed as focusing on team-directed reasoning. Nonetheless, my study refines a vital component of team reasoning.

**Figure 5.**
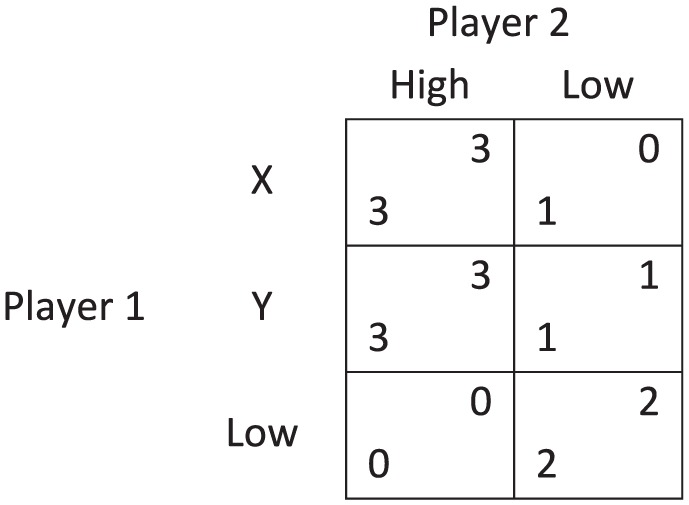
The ambiguous Hi-Lo game.

The team-reasoning schema can generally not be applied to typical collective action problems. To illustrate this defect and clarify the problem at this stage, consider the *ambiguous Hi-Lo game* depicted in Figure 5. It seems that a satisfactory theory of cooperation should recommend *high* to player 2 and should recommend that player 1 chooses either *X* or *Y*, rather than *low*. Team reasoning, unfortunately, fails to deliver either of these recommendations because it relies on the premise that there is a *unique* best group action available, as stated in premise (T6).^20^ In collective action problems, it is often the case that there are multiple ways to successfully coordinate the individual actions of the group members. For example, in committee decision making under the majority rule, it does not matter *who* votes for a particular option, it only matters that enough members vote accordingly. Similarly, in threshold public goods games, all strategy profiles in which sufficiently many individuals contribute is adequate. So team reasoning needs to be refined in order to apply it to collective action problems.

**Figure 6.**
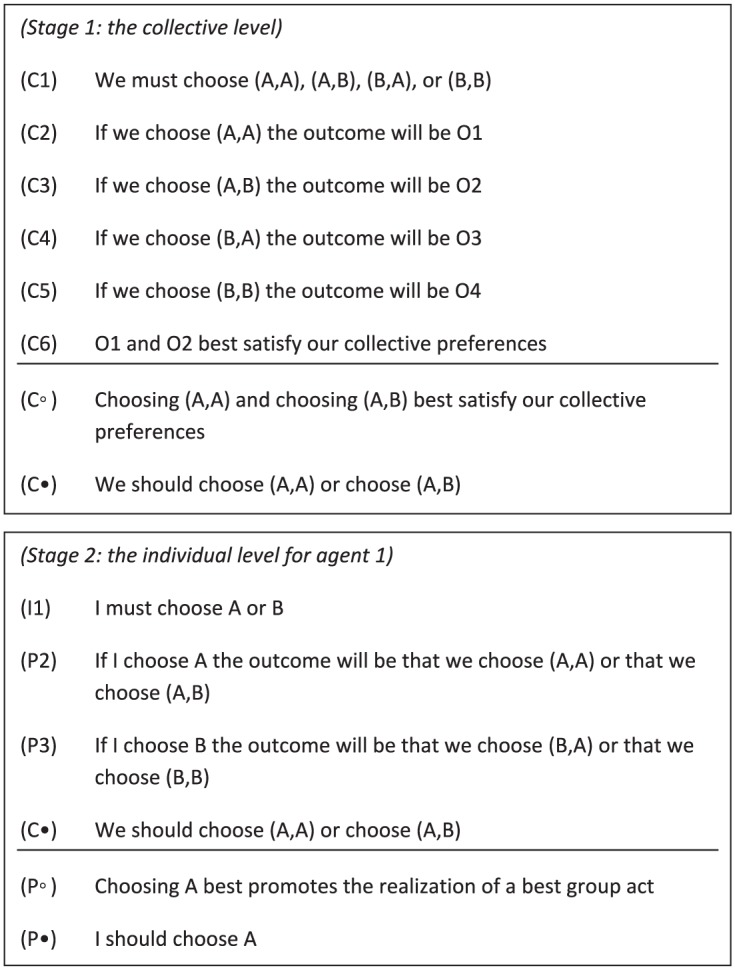
Participatory-reasoning schema: (P•) states the conclusion; (P◦), (C◦), and (C•) state intermediate conclusions; and the others state the premises.

To overcome this defect in team reasoning, I aim to contribute to theories of cooperation by augmenting the team-reasoning paradigm to what I call *participatory reasoning*. Roughly stated, participatory reasoning consists of two stages. First, a participatory-reasoner considers the group actions available to them, assesses these group actions in terms of their consequences, and finds the group actions that best further their common or collective interest. Second, a participatory-reasoner then considers the individual actions available to her, assesses these individual actions in terms of whether they yield a best group action, and then chooses an individual action that promotes the realization of a best group action. Accordingly, a member who endorses participatory reasoning can be thought of as aiming to participate in a best group action.^21^ If there are several best group actions, participatory reasoning can be viewed as yielding an individual action that maximizes the expectation of realizing a best group action. Figure 6 depicts the participatory-reasoning schema, which consists of two stages: the collective level and the individual level. That is, a participant first adopts the group perspective to answer the question “What should *we* do?” and then asks herself “What should *I* do?”

Can the participatory-reasoning schema do without the problematic uniqueness assumption? At the first stage, the collective-reasoning schema is adopted, which could yield a set of group actions. In the ambiguous Hi-Lo game, at the first stage a participant concludes that we should choose (*X, high*) or (*Y, high*). At the second stage, player 1 considers the options available to her and assesses the associated consequences. Since she seeks to realize a best group action, and because choosing *X* and choosing *Y* are both compatible with realizing a best group act whereas choosing *low* is not, participatory reasoning yields that she should either choose *X* or choose *Y*. This means that the participatory-reasoning schema can do without the problematic uniqueness assumption and can, therefore, be applied to typical collective action problems.^22^

Having refined team reasoning to participatory reasoning, let me end this section by addressing its main question: how do these practical-reasoning schemas relate to the distinction between competition and cooperation? To distinguish between the intentions that lie behind cooperative actions and the mutually consistent intentions that lie behind Nash equilibrium behavior, Natalie Gold and Robert Sugden (2007, 137) argue that

collective intentions are the product of a distinctive mode of practical reasoning, team reasoning, in which agency is attributed to groups.

Hence, whether a set of individual actions constitutes a collective intentional group action depends on the reasoning that led to the component individual actions. So what makes a set of individual actions a case of jointly intentional action is the form of reasoning endorsed by the members. Simply stated, if members endorse team reasoning, then the resulting pattern is a cooperative act, but if members endorse individual reasoning, then the resulting pattern is a mere aggregate of individual acts.

In cooperative settings, this means that the group members should endorse participatory reasoning rather than individual reasoning. Robert Sugden (1993, 72), for example, concurs:

A cooperative morality enjoins each individual to do her part in achieving outcomes that are good for all. . . . [T]he individual does not ask whether her own actions, considered in isolation, yield preferred outcomes.

This highlights that morality may even dictate which reasoning method is apt.^23^ Roughly stated, competitive contexts are characterized by the fact that individualistic reasoning is apt, and cooperative decision contexts are characterized by the fact that participatory reasoning is apt. This distinction will ground my analysis of potential responsibility voids.

## 5. Responsibility Voids Reconsidered

To distribute moral responsibility, my reasoning-based framework for moral responsibility highlights that it is useful to distinguish between two types of contexts: competitive and cooperative contexts. In other words, my framework distinguishes between endorsing individual or participatory reasoning. I will discuss these two cases in turn.

### 5.1. Competitive Decision Contexts

Do competitive decision contexts leave room for responsibility voids? As argued before, competitive decision contexts are characterized by the fact that the involved agents, or at least some of them, endorse the individual-reasoning schema. Stated boldly, I defend the claim that in a world of reasonable, rational individualists, there is no void between collective blameworthiness and the individual blameworthiness of its inhabitants. To be sure, there may be tragedy, but there is no void between, on the one hand, collective responsibility and, on the other hand, members’ responsibility.

It could be that all committee members in the discursive dilemma reasoned individualistically and that the premises they endorsed were fully justified. Does this leave a responsibility void with regard to the awarded tenure? There are two, jointly exhaustive, cases to distinguish. First, they could be justified in endorsing individualistic reasoning.^24^ This means that they reasonably faced a competitive problem. It is uncontroversial that competitiveness undermines group cohesion and group agency. According to most theories of moral responsibility, this would imply that the group cannot be attributed moral responsibility.^25^ Since this entails that the group is not *collectively* morally blameworthy for awarding tenure, it follows that there is no responsibility void. The result may certainly be a grave tragedy but not one in which there is some moral residue that should be distributed.

Second, they could be unjustified in endorsing individualistic reasoning. In this case, one could maintain that the group is collectively morally blameworthy for awarding tenure. For instance, the fact that the group forms a committee designed to decide on whether to award tenure may imply that the group is collectively morally blameworthy for the awarded tenure. On my reasoning-based framework of moral responsibility, this means that the committee members’ individual blameworthiness should be traced through the adopted reasoning methods. However, collective intentional group acts require that the members reason participatorily rather than individualistically. Hence, the faultiness is the absence of a collective intentional act, whose absence is constituted by the fact that some members reason individualistically. Therefore, any member who reasoned individualistically is an appropriate target of moral blame or sanction. That is, the group’s collective moral responsibility for the awarded tenure is distributed to those members who reasoned individualistically.

In either case, there is no responsibility void: competitive decision contexts are free from responsibility voids.

### 5.2. Cooperative Decision Contexts

Do cooperative decision contexts leave room for responsibility voids? As discussed before, cooperative decision contexts are characterized by the fact that the involved individuals endorse the participatory-reasoning schema. For simplicity’s sake, I assume that the decision context and the collective preferences are commonly known.^26^ To discuss potential responsibility voids, it is helpful to distinguish between two different types of uncertainty that the members of the group may jointly face. This distinction is natural if we note that the participatory-reasoning method treats expectations regarding external factors and in-group coordination differently. This difference gives rise to two types of uncertainty:

- *External Uncertainty*. In the first stage of participatory reasoning, group preferences over outcomes are lifted to preferences over group actions using the agent’s expectations regarding external factors. For example, these external factors may include what her opponents are doing, or it may concern physical properties of the world, such as whether it is currently raining.
- *Coordination Uncertainty*. In the second stage of participatory reasoning, preferences over individual actions are derived from the previously obtained best group actions and the agent’s expectations regarding individuals inside the group.

Both of these types of uncertainty may leave room for responsibility voids. However, the conditions for the existence of responsibility voids differ.

#### 5.2.1. External uncertainty

**Figure 7.**
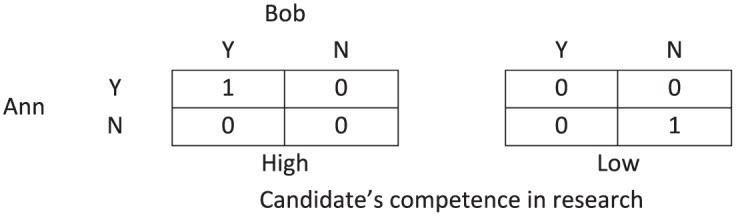
A two-player discursive dilemma, where the members need to decide to award tenure depending on the candidate’s excellence in research.

To illustrate the characteristics of cooperative decision contexts that include external uncertainty, it may be helpful to construct a simple game form. Imagine that two academics, Ann and Bob, form a committee that needs to decide on whether to award tenure to Mr. Borderline. This time, they do so by only judging the candidate’s excellence in research; say it is impossible for them to discuss this pre-vote or to adopt a premise-based decision procedure. The collective decision is based on unanimity, that is, the collective decision is *X* if and only if both of them voted for *X*. (This includes the possibility of collective indecision.)^27^ This case is depicted in Figure 7, where Ann and Bob need to vote “Y” or “N,” and the external factor is modeled as a third player. The group should coordinate on voting “Y” or on voting “N,” depending on whether the candidate is an excellent researcher. The best group action, therefore, depends on some external factor.

This case may uphold the possibility of a responsibility void. Such voids are possible if Ann and Bob have diverging beliefs regarding the candidate’s excellence in research. Say Ann thinks it is likely that the candidate is an excellent researcher whereas Bob thinks the opposite. Therefore, Ann will conclude, in the first stage of participatory reasoning, that they should coordinate on (Y, Y), whereas Bob will conclude that they should coordinate on (N, N). Then, in the second stage, each will reach the opposite conclusion, thereby yielding (Y, N): collective indecision results. Let us suppose that the candidate is an excellent researcher, yet he is not hired because of Ann and Bob’s collective indecision.

I will assume that the concept of collective moral responsibility makes sense in this case. Can any of the committee members be held responsible? The outcome results from the members’ diverging expectations regarding an external factor, the research excellence of the candidate in this case. This expectation affects the first stage of participatory reasoning in the premises concerning the expected outcomes of the available group acts, viz. (C2)–(C5). One of the committee members must be wrong in his or her expectation, and hence, in the absence of a plausible excuse, the member who has the least accurate expectations is most blameworthy; under the given circumstances, this would be Bob. So there would be no responsibility gap.

However, if Bob has reasonable or justifiable expectations, then he would not be responsible for the collective outcome. His mistake would then be justifiable. The study of these cases is beyond the scope of the current paper, and a discussion of whether certain expectations are reasonable or justifiable has to be left for future work.

Let me briefly clarify how this discussion can be transferred to the original discursive dilemma (Figure 1). Suppose the members award tenure in the way stated in the original formulation, but Mr. Borderline is actually a poor candidate. This means that the candidate is poor in at least one of the fields of competence. Let us say that the candidate is poor in research. In that case, participatory reasoning helps to clarify that the failure is in the premises (C2)–(C5). For instance, M1 would falsely think that the candidate is excellent in research and that they should decide correspondingly. Accordingly, the committee members who voted for the candidate’s excellence in research are most blameworthy; under the given circumstances, those are M1 and M3.

Although this brief discussion might not solve the responsibility distribution in cases of external uncertainty, it is important to note that the literature has largely neglected this type of uncertainty. Moreover, the literature on judgment aggregation (see List and Pettit 2011, chapter 2, for a useful overview) seems to largely focus on cases where the group jointly faces a decision problem under certainty. My discussion conveys the importance of cases of external uncertainty to debates on distributing responsibility.

#### 5.2.2. Coordination uncertainty

**Figure 8.**
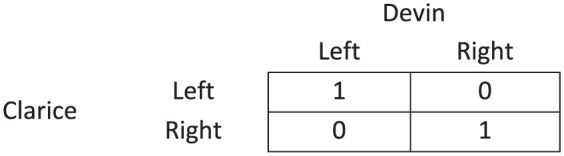
The footballers’ problem.

To illustrate the characteristics of cooperative decision contexts that include coordination uncertainty, it may be helpful to provide an informal example, which I will call the footballers’ problem, and to construct the associated game form.^28^ Suppose Clarice and Devin are two attackers of a football team. Clarice has the ball, but an opponent is approaching her. Devin, her team-mate, has more space, so she contemplates passing the ball to him. Let us suppose that there are two directions in which Devin could run, say, left and right. There are two corresponding points on the field, left and right, to which Clarice could pass the ball to be picked up by Devin. There is no time for communication, or for one player to wait and see what the other does: each must simultaneously choose left or right. Suppose that the move to the right puts Devin in an equally good position as the move to the left would. One could say that the probability that the pass will result in a goal if both choose right is the same as the probability if both choose left. If one chooses right and the other left, the probability is zero. Most importantly, this is a scenario in which the attackers must coordinate to solve the problem, there are two ways to solve the problem, and they are indifferent about which way they do it.^29^ This game is depicted in Figure 8, where Clarice and Devin need to choose *left* or *right*, and they need to coordinate their actions to have the best chance of scoring a goal.^30^ Let us suppose that they fail to coordinate and, therefore, fail to score a goal; as a result the team loses their match.

As noted before, I assume that collective moral responsibility makes sense in this case. That is, the team is collectively morally responsible for losing their match.^31^ Now, let us study whether this case could host responsibility voids, that is, the absence of individual moral responsibility.

When endorsing participatory reasoning, the first stage will result in the conclusion that Clarice and Devin should coordinate on either (*left, left*) or (*right, right*). This underdetermination may result in a responsibility void. Let us see how. Imagine that Clarice expects Devin to run *left*. At the second stage of participatory reasoning, she will think that her passing *left* is more likely to yield successful coordination, viz. (*P*2) and (*P*3). Consequently, she concludes that she should pass *left*. However, if Devin expects Clarice to pass *right*, he will, analogously, conclude that he should run *right*. Hence, the resulting group action will be (*left, right*), which is surely a suboptimal group act. In this case, it is impossible to point out who is at fault. Clarice may respond to Devin in two ways: “Why did you not *foresee* that I would pass *left*?” or “Why did you run *right*?” and hence, one blames the other for a wrong expectation or for a wrong choice. It is, however, unclear who is at fault: when Clarice blames Devin for not expecting her to pass *left*, Devin can react by blaming Clarice for not passing like he expected, that is, for failing to pass *right*.

Can any of the team members be held responsible? The outcome results from the members’ diverging expectations regarding in-group coordination, whether to coordinate on the left or on the right side of the field in this case. Their expectation affects the second stage of participatory reasoning in the premises concerning the expected outcomes of the available individual acts, viz. (*P*2) and (*P*3). In the absence of a coordinating mechanism, it is, however, impossible to say who has the least accurate expectation regarding coordination. A responsibility void arises.

How can we circumvent such a responsibility void? If communication is possible and agreement is unproblematic, then one way is to align the expectations of group members by letting them agree on a coordinating plan.^32^ Raimo Tuomela (2005, 345) writes,

If agreement making is in question, there will also be a publicly existing social (or, if you like, quasi-moral) obligation to participate in joint action. This entailment of an obligation can be regarded as a conceptual truth about the notion of agreement.

If communication is impossible, an alternative way to address this responsibility void is given by a theory of *salience*. In game theory, the most well-known discussion of salience is given by Thomas Schelling (1960, 57), who writes,

Most situations provide some clue for coordinating behavior, some focal point for each person’s expectation of what the other expects him to expect to be expected to do.^33^

A theory of salience may, therefore, highlight a particular strategy profile as salient. For example, in the footballers’ problem, it could be argued that in Sweden, the salient focal point is that both people coordinate on the right point on the field, that is, to coordinate on (*right, right*). It may thus be reasonable to expect that both are aware of this focal point. This means that members who have opposing expectations, and therefore aim at coordinating on (*left, left*), could be held responsible. The existence of a salient focal point would avoid the type of responsibility void just discussed.

Although agreements and focal points may circumvent this type of responsibility void, it remains unclear whether they are available in *every* cooperative decision context that includes coordination uncertainty. When these mechanisms are unavailable, my discussion reveals that responsibility voids potentially arise.

## 6. Conclusion

I have discussed the outline of a reasoning-based framework for moral responsibility and highlighted its application in collective action problems. The existence of responsibility voids depends on the nature of the decision context: competitive decision contexts are free of such voids, whereas cooperative decision contexts may host such voids. In the latter case, the conditions for the existence of these voids rest on the type of uncertainty the group faces, that is, either external or coordination uncertainty.

The common denominator in the conditions for the existence of responsibility voids is that they include justified false beliefs or expectations. The assessment of the legitimacy of these beliefs is hence vital for the study of responsibility voids. Let me briefly elaborate on cooperative decision contexts that include external uncertainty. I have argued that such cases may give rise to responsibility voids only if at least one of the group members has a false belief regarding some external factor and, furthermore, this false belief is justified. Practically speaking, if this situation arises in a case study, then one needs to determine whether the group members’ beliefs were justified *in that particular example*. For example, in the discussion of the two-player discursive dilemma (Figure 7), it is important to verify whether Bob’s false beliefs relied on culpable ignorance or negligence, which may, for instance, depend on whether he carefully read and checked the candidate’s CV.

In general, the importance of epistemic justification in collective action problems shows that theories of *expertise* and *disagreement* are relevant for the study of responsibility voids in democratic institutions. After all, democratic institutions often rely on committee decision making and expert opinions to deal with disagreement. I take it that an expert’s judgment is generally justifiable, whereas a layperson’s judgment is not. On the one hand, if the discursive dilemma is faced by experts, then it could be argued that each committee member’s belief is justified, regardless of whether it is correct, which implies that a responsibility void may result. On the other hand, if the discursive dilemma is faced by laypersons, then it could be argued that there are no responsibility voids. After all, in the absence of a justification for their false beliefs, laypersons can be held individually morally responsible for their contribution, and there is hence no responsibility void.

Responsibility voids have recently been used to justify the need for corporate responsibility and agency, as opposed to individual responsibility and agency, in order to dissolve the deficit in the moral accounting books. In other words, corporate responsibility is needed to address cases where no individual can be held responsible, yet “there is moral or rational fault that must be assigned somewhere” (Copp 2006, 216).^34^ If all of this is correct, then my analysis shows that the need for corporate responsibility and agency is only justified in cases that include justified false beliefs. Stated differently, justified false beliefs are a condition for corporate responsibility and agency. However, the justification for these false beliefs may need to be reconsidered in light of the prospect of responsibility voids and the relevance of such voids. In such a context, the demands for epistemic justification may be increased. Nonetheless, my analysis asserts that responsibility voids may exist. Hence, at least in some cases, there is a need for corporate responsibility.

## Footnotes

1 Smiley (2017) distinguishes between these three inquiries. I will assume that the concept of collective moral responsibility makes sense, at least in some cases. My argument in section 5.1 relies on the uncontroversial claim that competition undermines group agency.

2 Traditionally, decision theorists say that “we are in the realm of decision making under: 1. *Certainty* if each action is known to lead invariably to a specific outcome (the words prospect, stimulus, alternative, etc., are also used). 2. *Risk* if each action leads to one of a set of possible outcomes, each outcome occurring with a known probability. 3. *Uncertainty* if either action or both has as its consequences a set of possible specific outcomes, but where the probabilities of these outcomes are completely unknown or are not even meaningful” (Luce and Raiffa 1957, 13; emphasis added).

3 This is typically the subject of social choice theory. Sen, in his Nobel Prize lecture, says that the central question motivating social choice theory is this: “How can it be possible to arrive at cogent aggregative judgments about the society (for example, about ‘social welfare,’ or ‘the public interest,’ or ‘aggregate poverty’), given the diversity of preferences, concerns, and predicaments of the different individuals *within* the society?” (Sen 1999, 349).

4. List and Pettit (2002, section 4) discuss some, nonideal, strategies for dealing with the dilemma.

5. Although my perspective can be labeled as game-theoretical, it is important to stress that I plan to go beyond standard rational choice theory by endorsing the team-reasoning account of cooperation (Bacharach 2006; Sugden 2000).

6. To be explicit, Gold and Sugden (2007) present and discuss schemes for individual reasoning, collective reasoning, and several schemes for team reasoning. The participatory-reasoning schema (see section 4) is my novel addition to this range of practical-reasoning schemes.

7. The “I” is added to indicate that the statement occurs in the individual-reasoning schema. Similarly, for instance, a “C” will be added to statements that occur in the collective-reasoning schema (see Figure 3).

8. The example of the chess-player could be amended to prove this point: if the agent expects that two individual actions, say **e4** and **g6**, have the same chance of leading to victory, then individualistic reasoning would yield the recommendation to choose either of these.

9 I consider this indeterminacy an asset of the individual-reasoning schema. I agree with Bacharach (2006, 60, section 8.3) that a theory should only be determinate when our intuitions are. Compare Harsanyi and Selten (1988, 13): “Clearly a theory telling us no more than that the outcome can be any one of these equilibrium points will not give us much useful information. We need a theory selecting one equilibrium as the solution of the game.”

10 Unfortunately, I can only hint at how this could be done as a detailed explication would lead me too far astray.

11. Two of these concerns roughly correspond to what Braham and van Hees (2011) call “normative voids” and “epistemic voids.” My first concern has no counterpart in their theory.

12. Indeed, whether Malcolm actually made a *causal* contribution to Alice’s winning is irrelevant. It is only important that it played a role in his practical reasoning. Causality, however, is relevant when he assesses the available individual actions in terms of their consequences.

13 It should be clear from the above discussion that my view includes the possibility that the committee members try to “manipulate” the collective outcome by voting untruthfully. This may contradict one’s intuitions. However, for the assessment of moral responsibility, it is wholly unclear why truthful votes should take moral priority in interdependent decision problems. I do not wish to claim that every form of strategic decision making is morally acceptable—only some forms may be. I refer the interested reader to the paper by Dowding and Van Hees (2008) titled “In praise of manipulation.”

14 Bacharach (2006, chapter 1) and Sugden (2000, sections 2, 3, 7, and 8) argue that orthodox rational choice theory needs to be augmented with a collectivistic reasoning method to address certain cooperation problems—most notably, the so-called Hi-Lo game.

15. Important precursors within ethical theories of utilitarianism include those of Hodgson (1967) and Regan (1980). Later, Sugden (1991, 1993) fruitfully introduced team reasoning to the field of game theory. Similar ideas have been proposed by Bacharach (1999), Anderson (2001), Hurley (1989), and Gilbert (1989). Gold and Sugden (2007) and Hakli, Miller, and Tuomela (2010) have recently connected the team-reasoning literature to theories of collective intentionality.

16. In line with game-theoretical conventions, I use “(A,B)” to denote the strategy profile where the first player chooses A and the second player chooses B.

17 Gold and Sugden (2007, 123; terminology adapted) write, “We assert that [the individual-reasoning schema and the collective-reasoning schema] are both forms of valid instrumental reasoning and that they are valid for agents, defined as those entities that use these modes of reasoning (individual agents in one case, group agents in the other).”

18 There has been some discussion on the relation between group preferences and individual preferences. For example, Gold (2012, 195) writes that Bacharach “allowed in principle that the group objective might be welfare decreasing for some members,” and according to Sugden (2000, 176), “the preferences of a team are not necessarily reducible to, or capable of being constructed out of, the preferences that govern the choices that the members of the team make as individuals.” Nevertheless, Bacharach (1999, 120; terminology adapted) writes that, given a group of individuals *G*, “if it happens that [the group preferences are] Paretian, then it is good for everyone in *G* if everyone in *G* team reasons.”

19 Note that Sugden (2000, 195) writes, “Team-directed reasoning is something that one individual can engage in, independently of any others.”

20. Gold and Sugden (2007, 125), for example, write, “[T]he schema yields conclusions only when a profile that is the unique maximizer of the team payoff function exists.” In addition, Sugden (2000, 193) writes, “If two or more different combinations of strategies yield exactly the same utility for the team, this decision rule fails to determine what each individual should do.”

21 The participatory-reasoning schema intuitively relates to so-called participatory intentions, Kutz (2000, 81) writes, “Call this way of conceiving of action a participatory intention: an intention to do my part of a collective act, where my part is defined as the task I ought to perform if we are to be successful in realizing a shared goal. This conception of oneself as contributing to a collective, as manifested in one’s deliberation and action, is what lies at the heart of collective action generally, from simple coordination to complex cooperation.” I have studied the relation between team reasoning and participatory intentions in (Duijf, forthcoming).

22 It is important to investigate the conditions under which a member should/will team reason rather than reason individualistically. For my current purposes, it suffices to assume that team reasoning is eligible and justified in some collective action problems.

23 For instance, Regan (1980, 11) develops a theory of cooperative utilitarianism, which “can be summed up in the statement that each agent ought to co-operate, with whoever else is co-operating, in the production of the best consequences possible given the behavior of non-co-operators.”

24 For my arguments, it does not matter what grounds this justification.

25 There are few exceptions; Chant (2015, sections 4 and 5) argues against this position and proposes the “*revised agency thesis*: If moral responsibility attaches to an entity, then that entity must be an agent or a collective constituted by agents.”

26 Relaxing these conditions could reveal new ways in which responsibility voids may obtain. In accordance with my discussion of moral responsibility using the individual-reasoning schema in section 3, it may be helpful to say that these conditions entail that I only consider failures in premises (C2)–(C5), or (P2) or (P3), which describe the expected consequences of the available group acts and of the available individual acts, respectively.

27 Although the story is similar to the discursive dilemma, it should be clear that the structure of the decision context is different.

28 I have adapted the footballers’ problem from Sugden (2000, 179-80), who writes, “This problem is a simple model of a situation in which there is some objective (in this case, scoring a goal) which each of a set of individuals wants to achieve, and which can most effectively be achieved if those individuals coordinate their actions. Thus, we might say, it represents a situation in which social cooperation would be useful or valuable.”

29 This game can be interpreted as a coordination game (Lewis 1969).

30 One could say that Clarice and Devin have a shared intention to score a goal, which they can best realize by coordinating their individual actions. Bratman (1993, 99) writes, “Our shared intention performs at least three interrelated jobs: it helps coordinate our intentional actions; it helps coordinate our planning; and it can structure relevant bargaining.” The footballers’ problem shows that cooperation does not necessarily lead to successful coordination if communication is impossible.

31 The following discussion hence assumes that the group of footballers is collectively morally responsible *even though* communication is hampered. It is unclear whether such a scenario is universally accommodated by prominent accounts of collective action and responsibility. On the one hand, Kutz (2000, 92) writes, “My claim that nonresponsive or independent action can be jointly intentional does depend upon a disputable thesis: agents need not believe beforehand that they will be likely to succeed in their aims for their actions to count as jointly intentional.” On the other hand, Gilbert (2000, section 8.7; emphasis added) writes, “There is a group action if and only if the members of a certain population are jointly committed to pursuing a certain goal as a body, and in light of this joint commitment relevant members (perhaps not all) *successfully* act so as to *reach* the goal in question.”

32 In joint work with Allard Tamminga, I study how a group may regulate its group action by agreeing on a plan and then “characterize the conditions under which a group plan successfully coordinates the individual actions of the group members” (Tamminga and Duijf 2017, 187).

33 Schelling (1960, 57) thinks that these focal points may not be found within a theory of games: “Finding the key, or rather finding a key—any key that is mutually recognized as the key becomes the key—may depend on imagination more than on logic; it may depend on analogy, precedent, accidental arrangement, symmetry, aesthetic or geometric configuration, casuistic reasoning, and who the parties are and what they know about each other.”

34 The following observation does not affect the argument by Pettit (2007, 175) that corporations can meet the three conditions for fitness to be held responsible, namely “value relevance,” “value judgment,” and “value sensitivity.”
